# Prevalence of integrons 1, 2, 3 associated with antibiotic resistance in *Pseudomonas aeruginosa* isolates from Northwest of Iran

**DOI:** 10.1051/bmdcn/2018080102

**Published:** 2018-02-26

**Authors:** Shahram Mobaraki, Mohammad Aghazadeh, Mohammad Hossein Soroush Barhaghi, Mohammad Yousef Memar, Hamid Reza Goli, Pourya Gholizadeh, Hossein Samadi Kafil

**Affiliations:** 1 Drug Applied Research Center, Tabriz University of Medical Sciences Tabriz Iran; 2 Iranian Center of Excellence in Health Management, Tabriz University of Medical Sciences Tabriz Iran; 3 Biotechnology Research Center, Tabriz University of Medical Sciences Tabriz I.R. Iran; 4 Department of Medical Microbiology and Virology, Faculty of Medicine, Mazandaran University of Medical Sciences Sari I.R. Iran; 5 Student Research Committee, Tabriz University of Medical Sciences Tabriz I.R. Iran; 6 Hematology and Oncology Research Center, Tabriz University of Medical Sciences Tabriz I.R. Iran

**Keywords:** Pseudomonas aeruginosa, Antibiotic resistance, Integrons, Iran

## Abstract

Background: The presence of Class 1, 2 and 3 integrons in clinical isolates of *Pseudomonas aeruginosa* with multi-drug resistance phenotype has rendered the organism as a new concern. Objective: This study aimed to investigate the prevalence of Class 1, 2 and 3 integrons in multi-drug resistant clinical isolates of *Pseudomonas aeruginosa* collected from hospitals in the city of Tabriz

Materials and Methods: A total of 200 *P. aeruginosa* non-duplicated clinical isolates were collected from inpatients and outpatients in different wards of hospitals from May to November 2016. The bacteria were identified by conventional microbiological methods. Antibiotic susceptibility test was performed by disk diffusion method and the presence of integrons was analyzed by polymerase chain reaction (PCR).

Results: Colistin was the most effective antibiotic, while 98% of the isolates were resistant to cefotaxime. Fifty-three percent of the isolates were recorded as multi-drug resistant (MDR) phenotype; however, 27.5% of the isolates were resistant to more than 8 antibiotics. In this study, 55 (27.5%), 51 (25.5%), and 30 (15%) clinical isolates of *P. aeruginosa* were positive for Class 1, 2 and 3 integrons, respectively. *aac(6)*II in Class I integrons and *dfrA1* in ClassII and *aacA7* in Class II integrons were the most prevalent genes. Resistance to aminoglycosides were the most common genes harbored by integrons.

Conclusion: The results of this study showed that the prevalence of Class 1, 2 and 3 in integron genes in most *P. aeruginosa* strains islated from different parts and equipment used in the hospital. The role of these transferable genetic agents has been proven in the creation of resistance. Therefore, it is essential to use management practices to optimize the use of antibiotics, preferably based on the results of antibiogram and trace coding genes for antibiotic resistance.

## 1. Introduction

*Pseudomonas aeruginosa* is a common environmental, Gramnegative, ubiquitous bacterium that causes a variety of infections in immunocompromised, hospitalized patients [1, 2]. This organism trends to increase resistance towards many antimicrobial agents and a high percentage of the *P. aeruginosa* clinical isolates show the multidrug resistance (MDR) phenotype [3, 4]. The most effective anti-pseudomonal agents are beta-lactams, aminoglycosides and fluoroquinolones [5, 6]. Mechanisms of resistance to antimicrobial agents include production of beta-lactamases, multidrug efflux pumps, presence of integrons and downregulation of outer membrane porins [5-7]. Many of the antibiotic resistance genes found on plasmids and transposons that are located at a unique site named integron [8, 9]. These elements mediate the integration of genes through the action of a DNA integrase (*int*I) and a specific recombination site (*att*I) that acts as a receptor of gene cassettes [10]. Approximately, There are 90 distinct integron Classes that most of them located on chromosomes, and about 10% of the sequenced bacterial genomes carry these elements [11]. The first of integrons that have been described are Classes 1, 2, and 3 that exhibit a number of features not typical of the more numerically dominant chromosomal integron Classes. In total, they are carried on transposons and/or plasmids and most commonly contain up to 6 cassettes drawn from a pool of about 100 cassettes and almost all of which encode antibiotic resistance determinants [12, 13]. Class 1, 2 and 3 are three main well characterized integrons [14, 15]. The Classe 1 integrons are the most common integrons that found in *P. aeruginosa, Acinetobacter baumannii* and in members of *Enterobacteriaceae* family [16, 17]. Prevalence of Class 2 and 3 integrons among these pathogens is not widely reported [18, 19]. The aim of this study is the analysis of Class 1, 2 and 3 integrons prevalence of and their association with drug resistance in clinical isolates of *P. aeruginosa.*

## 2. Materials and Methods

### 2.1. Bacterial isolates and clinical data

We collected 200 non-duplicated *P. aeruginosa* clinical isolates from inpatient and outpatients in three teaching and treatment hospitals of Tabriz, Iran from 2015 to 2016 (September to April). The isolates were obtained from blood, sputum, urine, respiratory tract, wound, and cerebral spinal fluid (CSF). The bacteria were identified by routine microbiological tests such as Gram stain, inability to fermentation of lactose, oxidation and fermentation test (O/F), oxidase test, growth on Cetrimide agar medium (Liofilchem, Italy), pigmentation test [20].

### 2.2. Antimicrobial susceptibility testing

The antibiotic susceptibility test was done by Kirby-Bauer disk diffusion method according to the Clinical and Laboratory Standards Institute (CLSI) guidelines. The antibiotic disks (MAST, England) included amoxicillin-clavulanate (20/10 μg), imipenem (10 μg), colistin (10 μg), amikacin (30 μg), cefepime (30 μg), cefotaxime (30 μg), ceftazidime (30 μg), tobramycin (30 μg), gentamicin (30 μg), ciprofloxacin (5 μg), Polymyxin B (300 units), gatifloxacin (5 μg) and piperacillin (100 μg) [21, 22]. MDR was definite as acquired resistant to at least one agent in three or more antimicrobial Classification [23]. *P. aeruginosa* ATCC 27853 was used as quality control strain [21].

### 2.3. DNA extraction and detection of int genes

Genomic DNA was extracted by the tissue buffer boiling method. Precisely, similar colonies of the bacterial isolates were mixed with 20 μ1 of tissue buffer (0.25% SDS + 0.05 M NaOH) and the mixture was incubated for 15 minutes in 95°C [24]. The mixture was centrifuged for 1 minute in 13,000 g. After centrifugation, 180 ìl of Milli-Q water was added to the aqueous solution. The extracted DNA was frozen in -20°C until usage [25].

The *int* genes were amplified by the polymerase chain reaction (PCR) method. Each PCR reaction was done by CINNAGEN master mix (SinaClon, Tehran, Iran) and specific primers which are shown in Table 1. The amplification was carried out in a DNA thermal cycler (Eppendorf master cycler gradient, Germany) as follows: initial denaturation at 94°C for 10 min, followed by 30 to 40 repeated cycles of denaturation at 94°C for 40 s, 50 s for annealing at 57°C for *int*I-1, *int*I-2 and 59°C for *int*I-3, and 55 s for extension at 72°C, followed by 10 min at 72°C for final extension. The amplified products were analyzed by electrophoresis on 1% agarose gel and staining by the ethidium bromide [26].

**Table 1:** List of primers were used for the PCR amplification and sequencing of integrons in the present study.

Target region	Primer sequence (5́ → 3́)	Size of product	Annealing Temperature	References
*int*I-1F	TCATGGCTTGTTATGACTGT GTAGGGCTTATTATGCACGC	600 bp*	57̊C	26
*int*I-1R				
*int*I-2F	GATGCCATCGCAAGTACGAG CGGGATCCCGGACGGCATGCACGATTTGTA	750 bp*	57̊C	27
*int*I-2R				
*int*I-3F	GCCTCCGGCAGCGACTTTCAG ACGGATCTGCCAAACCTGACT	650 bp*	59̊C	28
*int*I-3R				

* bp = base pair, F = forward sequence, R = reverse sequence.

### 2.4. Statistical analysis

SPSS Version 22 (IBM SPSS Statistics, New York, USA) was used for statistical analysis. Descriptive statistics, Chi-square or Fisher's exact test was used to evaluate the data. *P*-value below 0.05 was considered statistically significant.

## 3. Results

Among 200 *P. aeruginosa* isolates, 145 of them were collected from Imam Reza hospital, while 35 and 20 isolates were obtained from Sina and Pediatric hospital, respectively. Also, 115 isolates were from inpatients and others were achieved from outpatients. Fifty percent of the patients were men and the age ranges of patients were from new-born to 89 year-old. The clinical isolates were collected from sputum (3 isolates), CSF (7 isolates), purulent wound (57 isolates), blood (19 isolates), respiratory tracts (38 isolates), and urine (76 isolates).

The results of antibiotic susceptibility test were shown in Table 2. The isolates were most sensitive to polypeptide antibiotics such as Colistin. The results showed that the majority of the isolates (98%) were resistant to Cefotaxime (Table 2) and 53% of the isolates were multidrug resistance (MDR).

**Table 2:** The relationship between the presence of integrons and resistance to antibiotics in clinical isolates of Pseudomonas aeruginosa in the present study.

	All isolates (n = 200)			Integron positives (n = 55)			Integron negatives (n = 145)			
				
	
Antibiotics	R*	I*	S*	R*	I*	S*	R*	I*	S*	*P* value**
	No. (%)	No. (%)	No. (%)	No. (%)	No. (%)	No. (%)	No. (%)	No. (%)	No. (%)	
Amikacin	110	9	81	34	2	19	79	3	63	NS*
	(55)	(4.5)	(40.5)	(61.8)	(3.6)	(34.5)	(54.4)	(2)	(43.4)	
Cefepime	125	0	75	36	0	19	89	0	56	NS
	(62.5)		(37.5)	(65.4)		(34.5)	(61.3)		(38.6)	
Ceftazidime	113	0	67	40	0	15	93	0	52	NS
	(66.5)		(33.5)	(72.7)		(27.2)	(64.1)		(33.8)	
Tobramycin	115	0	85	26	0	29	86	0	59	NS
	(57.5)		(42.5)	(47.2)		(52.7)	(59.3)		(40.6)	
Gentamicin	124	0	76	39	0	16	85	0	60	NS
	(62)		(38)	(70.9)		(29)	(58.9)		(41.3)	
Imipenem	93	12	95	25	3	17	73	5	67	NS
	(46.5)	(6)	(47.5)	(45.4)	(5.4)	(30.9)	(50.3)	(3.4)	(46.2)	
Colistin	6	0	194	2	0	53	4	0	141	NS
	(3)		(97)	(3.6)		(96.3)	(2.7)		(97.2)	
Ciprofloxacin	125	0	75	33	0	22	88	0	57	NS
	(62.5)		(37.5)	(60)		(40)	(60.6)		(39.3)	
Amoxicillinclavulanate	121	0	79	33	0	22	86	0	59	NS
	(60.5)		(39.5)	(60)		(40)	(59.3)		(40.6)	
Cefotaxime	149	3	48	39	1	15	109	2	34	NS
	(74.5)	(1.5)	(24)	(70.9)	(1.8)	(27.2)	(75.1)	(1.3)	(23.4)	
Ceftazidimeclavulanate	110	0	90	33	0	22	77	0	68	NS
	(55)		(45)	(60)		(40)	(53.1)		(46.8)	

* R: resistant. I: intermediate. S: susceptible. NS: not statistically significant.

** Statistical analysis was done by *Chi*-square method using SPSS software. All other statistical analysis were done by descriptive methods.

**Table 3:** Gene cassettes in the Class I integrons in clinical isolates of *Pseudomonas aeruginosa*.

Length of variable region (s) (bp)	Gene cassette (s)	No. of isolates (%)	The name of hospital (s)
750	*aadB*	3 (5.4%)	I,I,I
1200	*aadA6-orfD*	4 (7%)	I,I,I,I
1200	*aadB-aadA1, blaoxa-10+* *(aac(6)-II), blaoxa-10*	13 (24%)	I,I,I,I,S,S,I,S,S,I,I,I,I
1250	*aac(6)-II, aacA4,* *blaoxa-10+blavlm-6,* *aac(6)-Ib*	20 (36%)	I,I,I,I,I,S,I,I,I,I,I,I,I,S,S,S,I,I,I,I
1500	*aacA4-catB10*	2 (3.5%)	I,I
1700	*aacA4-bla*_*OXA10*_	13 (24%)	I,I,I,I I,I,I,I,I,I,I,I,I,I
Total		**55 isolates**	

* I: Imam, S: Sina. C: Kodakan.

**Table 4:** Gene cassettes in the Class II integrogens structure by PCR method in clinical isolates of *Pseudomonas aeruginosa*.

Length of variable region (s) (bp)	Gene cassette (s)	No. of isolates (%)	The name of hospital (s)
500	*dfrA1*	15 (30%)	I,I,I,I,I,S,S,S,I,I,S,I,I,I,P
600	Hypothetical gene cassette	15(30%)	I,I,I,I,I,I,I,S,S,P,P,S,S,I,I
Total		**30 isolates**	

*I: Imam. S: Sina. C: Kodakan.

**Table 5:** Gene cassettes in the Class III integrogens structure by PCR method in clinical isolates of *Pseudomonas aeruginosa*.

Length of variable region (s) (bp)	Gene cassette (s)	No. of isolates (%)	The name of hospital (s)
400	*aacA7+ aacA4-blaoxA2*	30(59%)	I,I,I,I,I,I,I,S,S,S,I,I,I,I,I,I,I,S,I,S,I,I,I,I,P,I,I,I,I,I
600	Hypothetical gene cassette	21(41%)	I,I,I,S,I,I,S,I,I,I,I,S,I,I,I,P,S,P,P,S,P
Total		**51 isolates**	

* I: Imam. S: Sina. C: Kodakan.

All isolates were evaluated with PCR analysis to detect integrons Classes. According to the PCR results, 55 (27.5%), 51 (25.5%), and 30 (15%) isolates were contained Class 1, 2, and 3 integron, respectively. The results of association between antibiotic resistance and presence of the integrons are shown in Table 3, 4 and 5. In this study, there was no significant association between antibiotic resistance and the presence of integron among the isolates.

**Fig. 1:**
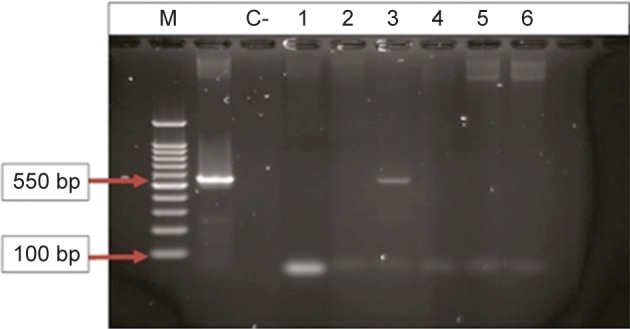
PCR product of the amplification of *int*-1after agarose electerophoresis. M: ladder (100 bp). C+: control positive of a gene (*int*-1). C-: control negative. 1-6: *Pseudomonas aeruginosa* isolates (sample number 3 was positive).

## 4. Discussion

*P. aeruginosa* is an opportunistic pathogen that encompasses a wide range of human infection [4], particularly resistant to many antibiotics that makes it hard to treat [27]. Currently it is known one of the most important nosocomial infections with high mortality. Recent studies have shown that transfer of resistance genes by integrons has important role in acquiring resistant in bacteria. Many resistance genes can be transferred by integrons. These genes can be originated by plasmids and transposons [18]. This study aimed to track presence of three main Classes of integrons including Class 1, 2, 3 integrons in *P. aeruginosa* isolated from main hospitals in northwest of Iran. The most prevalent integron in our isolates was Class 1 with presence in 55% of isolates. However, 78.4% of isolates with Class 2 integrons were MDR, which shows its importance in transfer of resistance genes. All identified integrons harbored gene cassettes and *aad* and *aac* genes were the most prevalent genes in our isolates. These genes are corresponding to resistance to aminoglicosides. Several recent studies reported high presence of integrons harboring resistance genes cassettes such as Ren *et al.* in 2012 from the United States[28], Kali *et al.* In 2011, from France [29], Tacon *et al.* In 2012 from Brooklyn [30], as well as Taghavi *et al.* in 2013 from Iran [31]. In our study, most of isolates were isolated from infected wounds (28.5%) and urinary tract infection (38%), respectively. In Babay study in Saudi Arabia, the most prevalence of isolates from wounds was reported [32]. Antibiotic susceptibility pattern of *P. aeruginosa* showed 53% of isolates were resistant to more than 5 antibiotics. In Thailand, Poonsuk *et al.* showed an increase in resistance of *P. aeruginosa* isolates to Amikacin (92.1%), Ceftazidime (96%), Gentamicin (99%) and Cipro-floxacin (95%)[33]. Fazeli *et al.* have shown that *P. aeruginosa* isolates were resistant to Ciprofloxacin (29%) and Gentamicin (32.2%) [34]. Ciprofloxacin is one of the best options available for the treatment of infections caused by *P.aeruginosa*, particularly in treatment of urinary tract infections [35]. In our isolates, 62.5% of isolates were resistant to Ciprofloxacin. In Latin America (26.8%) and Europe (32%) of isolates were reported to be resistant to Ciprofloxacin [36-38]. In our study, frequency of Class 1, 2 and 3 integrons were 55 (27.5%), 51 (25.5%), and 30 (15%) of isolates, respectively. Other studies from our country reported Class 1 Integrons in 95% of the isolates and Class 2 in 54% and Class 3 in 10% of the isolates [39]. In a study by Shibata *et al.*, integron 1 was the most common integron and integron 3 was observed sporadic in isolates from Japan [40]. Integrons 2 is reported from 9% of isolates from Zanjan- Iran [41] and was reported in 5.3% of isolates isolated from Malaysia [42]. In the present study, Colistin was the most effective antibiotic against *P. aeruginosa* (Table 2). Highest resistance was observed to cefotaxime (98%). All of the integron positive isolates in the present study Contained genetic cassettes. *aad* and *aac* genes family were the most common genes in cassettes. These genes are corresponding on resistance to aminoglycosides. In a same study conducted in neighbor region of our country (Turkey) the most common gene in cassettes was *aad* gene. In the present study *aac* (6)–II gene was the most common gene identified in the cassettes. This gene is the most common identified gene in the structure of Class 1 integrons, in clinical isolates of *P.aeruginosa* reported worldwide. This gene (*aac* (6) –II) is an enzyme encoding an aminoglycoside ('6) *-N-acetyltransferase (II- (6) AAC)*, which causes resistance to Netilmicin, tobramycin and kanamycin. *aadB* gene was the second common genes in our studied cassettes. This gene codes the enzyme aminoglycoside ("2) - adenyltransferase (ANT (2") - Ia), which can cause resistance to kanamycin, gentamicin and tobramycin. The *aadA1* and *aacA4* genes were other identified genes and blaoxa-10 gene from Class D of bosh penicillinase, as a broad-spectrum beta-lactamase. This gene is corresponding to enzymes to hydrolyze the beta-lactam antibiotics such as penicillins. In addition, in several isolates we had co-presence of *aacA4* and *cat*B 10 genes ,which is only reported previously from isolates originated from Iran [43]. The *cat*B 10 gene is a chloramphenicol acetyltransferase enzyme causes resistance to chloramphenicol. Blast of gene cassettes in our isolates showed presence of two new genes in cassettes including blaoxa-10+blavlm-6 and *aac*(6)-Ib which there is no previous report for them from Iran. Presence of resistance in isolates and their easy movement indicates importance of infection control and stewardship programs in hospitals [44]. In this study, there was no significant association between antibiotic resistance and the presence of integron among the isolates. It may be due to a high rate of resistance in our isolates and a low number of sensitive isolates or vis-versa. But future studies the same number of resistant and sensitive isolates can be more helpful to define they exact role on resistance. The finding of the present study is a comprehensive study on integron carriage in our study region and will help clinicians to define the best stewardship for controlling distribution of the resistance.

## 5. Conclusion

Results of the present study indicate increasing prevalence of integrons corresponding to antibiotic gene cassettes movement. These integrons had genes for resistance to different family members and caused multi drug resistance in our isolates. We had less prevalence of class 1 integrons but higher prevalence of class 3 integrons. Presence of different resistance genes indicates high risk of resistance transmission and distribution of MDR isolates in hospitals. Antibiotic consumption control and antibiotic stew-warship are necessary for reducing resistance in clinical isolates in this region.

## Acknowledgments

This study was supported by Drug Applied Research Center, Tabriz University of Medical Sciences, as the Master's thesis of Mr Mobaraki. We thank all hospital staff for their collaboration in sample collection.
